# Fibrosis, Connexin-43, and Conduction Abnormalities in the Brugada Syndrome

**DOI:** 10.1016/j.jacc.2015.08.862

**Published:** 2015-11-03

**Authors:** Koonlawee Nademanee, Hariharan Raju, Sofia V. de Noronha, Michael Papadakis, Laurence Robinson, Stephen Rothery, Naomasa Makita, Shinya Kowase, Nakorn Boonmee, Vorapot Vitayakritsirikul, Samrerng Ratanarapee, Sanjay Sharma, Allard C. van der Wal, Michael Christiansen, Hanno L. Tan, Arthur A. Wilde, Akihiko Nogami, Mary N. Sheppard, Gumpanart Veerakul, Elijah R. Behr

**Affiliations:** ∗ Pacific Rim Electrophysiology Research Institute, Los Angeles, California; † Cardiovascular Sciences, St. George's, University of London, London, United Kingdom; ‡ Centre for Translational & Experimental Medicine, Imperial College London and Hammersmith Hospital, London, United Kingdom; § Department of Molecular Physiology, Nagasaki University Graduate School of Biomedical Sciences, Nagasaki, Japan; ‖ Department of Heart Rhythm Management, Yokohama Rosai Hospital, Yokohama City, Japan; ¶ Bhumibol Adulyadej Air Force Hospital, Royal Thai Air Force, Bangkok, Thailand; # Department of Pathology, Siriraj Hospital, Mahidol University, Bangkok, Thailand; ∗∗ Heart Centre, Academic Medical Centre, Amsterdam, the Netherlands; †† Clinical Biochemistry, Statens Serum Institute, Copenhagen, Denmark; ‡‡ Princess Al-Jawhara Al-Brahim Centre of Excellence in Research of Hereditary Disorders, Jeddah, Saudi Arabia; §§ Cardiovascular Division, Faculty of Medicine, University of Tsukuba, Tsukuba, Japan

**Keywords:** gap junction, myocardial fibrosis, right ventricular outflow tract, sudden arrhythmic death syndrome, sudden unexpected death, BrS, Brugada syndrome, Cx43, connexin-43, ECG, electrocardiogram, ICD, implantable cardioverter-defibrillator, LV, left ventricle/ventricular, OR, odds ratio, PSR, picrosirius red stain, RV, right ventricular, RVOT, right ventricular outflow tract, SADS, sudden arrhythmic death syndrome, SCD, sudden cardiac death, SCN5A, sodium channel, voltage gated, type V alpha subunit, VT, ventricular tachycardia, VF, ventricular fibrillation

## Abstract

**Background:**

The right ventricular outflow tract (RVOT) is acknowledged to be responsible for arrhythmogenesis in Brugada syndrome (BrS), but the pathophysiology remains controversial.

**Objectives:**

This study assessed the substrate underlying BrS at post-mortem and in vivo, and the role for open thoracotomy ablation.

**Methods:**

Six whole hearts from male post-mortem cases of unexplained sudden death (mean age 23.2 years) with negative specialist cardiac autopsy and familial BrS were used and matched to 6 homograft control hearts by sex and age (within 3 years) by random risk set sampling. Cardiac autopsy sections from cases and control hearts were stained with picrosirius red for collagen. The RVOT was evaluated in detail, including immunofluorescent stain for connexin-43 (Cx43). Collagen and Cx43 were quantified digitally and compared. An in vivo study was undertaken on 6 consecutive BrS patients (mean age 39.8 years, all men) during epicardial RVOT ablation for arrhythmia via thoracotomy. Abnormal late and fractionated potentials indicative of slowed conduction were identified, and biopsies were taken before ablation.

**Results:**

Collagen was increased in BrS autopsy cases compared with control hearts (odds ratio [OR]: 1.42; p = 0.026). Fibrosis was greatest in the RVOT (OR: 1.98; p = 0.003) and the epicardium (OR: 2.00; p = 0.001). The Cx43 signal was reduced in BrS RVOT (OR: 0.59; p = 0.001). Autopsy and in vivo RVOT samples identified epicardial and interstitial fibrosis. This was collocated with abnormal potentials in vivo that, when ablated, abolished the type 1 Brugada electrocardiogram without ventricular arrhythmia over 24.6 ± 9.7 months.

**Conclusions:**

BrS is associated with epicardial surface and interstitial fibrosis and reduced gap junction expression in the RVOT. This collocates to abnormal potentials, and their ablation abolishes the BrS phenotype and life-threatening arrhythmias. BrS is also associated with increased collagen throughout the heart. Abnormal myocardial structure and conduction are therefore responsible for BrS.

Brugada syndrome (BrS) is an inherited arrhythmia syndrome diagnosed by the presence of the type 1 Brugada electrocardiogram (ECG) (1). It was initially described in survivors of cardiac arrest without structural disease (2), and it is partly responsible for sudden arrhythmic death syndrome (SADS) 1, 3, 4. Potential causal variants in the cardiac sodium channel gene *SCN5A* are identified in 20% of cases (5). It was initially proposed that the basis for BrS was an abnormal transmural repolarization in the right ventricular outflow tract (RVOT) due to heterogeneous loss of the cardiomyocyte action potential dome in the epicardium (6). However, electrophysiological, imaging, and histopathological studies have identified subtle structural abnormalities in patients with BrS 7, 8, 9. Myocardial fibrosis has been suggested by abnormal, low-voltage, fractionated electrograms localized to the RVOT at the epicardium 9, 10. Ablation at these sites has eliminated the type 1 Brugada ECG pattern and successfully reduced arrhythmic events (10), as was seen in a previous experimental model (11).

A study of sudden cardiac death (SCD) cases associated the type 1 ECG with arrhythmogenic right ventricular cardiomyopathy (8). Furthermore, SCD cases with a familial diagnosis of BrS showed structural abnormalities that were insufficient to fulfill the diagnostic criteria for cardiomyopathy or myocarditis (12). Other myocardial anomalies have been reported in selected cases 13, 14. Therefore, there is significant debate about the underlying substrate in BrS (15).

To resolve this controversy, we tested the hypothesis that BrS is associated with fibrosis in the RVOT and altered expression of the gap junction protein connexin-43 (Cx43), which may be critical for correct cellular migration and maintenance of RVOT zonation 16, 17. We expected this to manifest as abnormal late and fractionated potentials at the RVOT epicardium.

## Methods

### Study setting and cohorts

#### Post-mortem BrS cohort

From 2005 to 2010, 1,304 unexpected SCD cases were referred for specialist cardiac autopsy. We studied 6 male cases (B1 to B6; mean age 23.2 years) (Table 1), which fulfilled the following criteria for SADS (1): 1) age 1 to 64 years; 2) unexpected sudden death; 3) whole heart available; 4) heart morphologically normal at coronial/medical examiner and specialist cardiac autopsies; 5) no antemortem cardiac conditions; and 6) negative toxicological analysis. In addition, 1 or more first-degree blood relatives had to be diagnosed with BrS (Online Methods) following familial evaluation 1, 18, 19.

All 6 cases were asymptomatic before death, according to primary care records and family interview, with no family history of premature death. Five died at rest (4 during sleep) and 1 during exertion. None had undergone previous cardiac investigation.

#### Post-mortem control cohort

Six control cases (C1 to C6) (Table 1) of premature noncardiac death were identified from 407 consecutive homograft valve donors from Harefield Hospital, London (2010 to 2012). These were matched to the post-mortem BrS cases by random risk set sampling selection for age (within 3 years) and sex in a 1:1 ratio. Inclusion criteria for control cases were: 1) age 1 to 64 years; 2) absence of antemortem cardiac symptoms (syncope or seizures); 3) normal specialist cardiac autopsy; and 4) intact RVOT.

#### In vivo BrS ablation cohort

Six symptomatic male BrS patients (mean age 39.8 years) (Table 1) undergoing mapping and RVOT ablation during open thoracotomy were studied at Bhumibol Adulyadej Air Force Hospital (cases V1 to V5, Bangkok) and Yokohama Rosai Hospital (case V6, Japan). All had an implantable cardioverter defibrillator (ICD) before recruitment, with a clinical diagnosis (Online Methods) of BrS 1, 19, and normal echocardiography, computed tomography/magnetic resonance imaging, and coronary angiography. Thoracotomy was indicated for ICD lead extraction (V1, V2, V5, and V6) or to permit epicardial access for ablation after a failed percutaneous attempt (V3 and V4).

### Mutation analysis

In vivo BrS subjects and clinically affected blood relatives of post-mortem cases were counseled and offered *SCN5A* mutation analysis. Mutation analysis was not undertaken in the autopsy cases due to lack of suitable unfixed material.

### Specialist cardiac post-mortem examination

A systematic specialist post-mortem of the whole heart was undertaken, with macroscopic and microscopic evaluation in all referred SCD cases and control hearts, blinded to the results of familial evaluation (20). At least 20 tissue sections were sampled from each case, including the following: coronary arteries; ascending aorta; 4 sequential sections from the atrioventricular node to the branches of the His-Purkinje system; 4 sinoatrial node sections; and 2 RVOT sections. Sectioning of the anterior, lateral, and posterior left ventricle (LV), anterior and posterior interventricular septum, and right ventricle (RV) was performed at the midventricular level. Histological examination (Online Methods) was performed with hematoxylin and eosin and elastic Van Gieson stains.

### Detailed post-mortem RVOT examination

Up to 14 parallel longitudinal sections of 3-mm thickness were taken from the RVOT in each post-mortem subject to ensure complete examination of this region.

#### Morphometric analysis for post-mortem myocardial collagen/fibrosis

**Figure 1:**
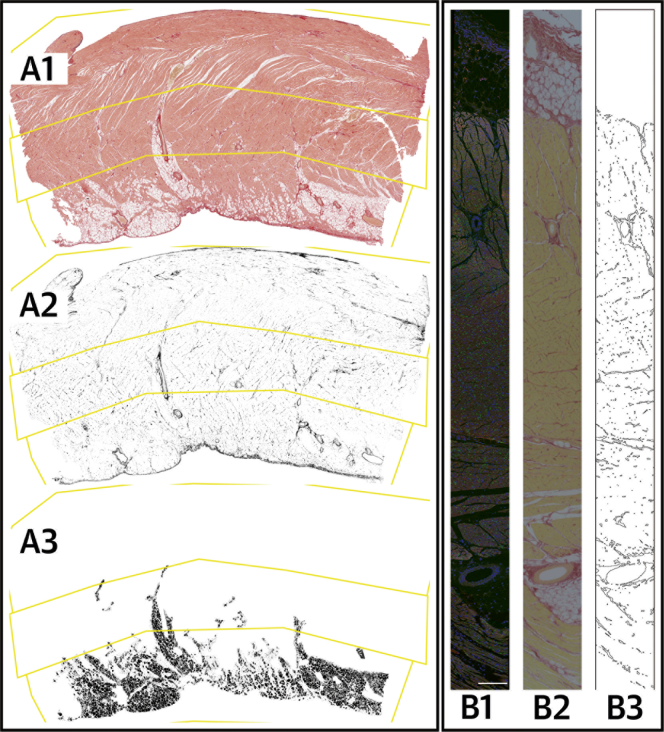
Morphometric Analysis of Histological Stained Sections **(A)** Morphometric analysis of a single tissue section. **(A1)** Visually defined tissue zones (**yellow** polygons) defining the epicardium, mid-myocardium, and endocardium. **(A2)** Collagen **(black)**. **(A3)** Fat cells **(black)**. **(B)** Representative serial myocardial strips from the post-mortem control group for collagen correction for Cx43 morphometric analysis. **(B1)** Myocardial strip of Cx43 expression. **(B2)** Serial section aligned to B1, stained with PSR. **(B3)** A threshold drawing generated from the PSR-stained myocardium image (as created by ImageJ). Scale bar = 200 μm; Cx43 = connexin-43; PSR = picrosirius red.

All post-mortem RVOT sections were stained with the picrosirius red (PSR) technique, with RV free wall and LV tissue for comparison. These sections (n = 267, total area quantified 6,505 mm^2^) were digitized (Scanscope CS, Aperio, California) at 20× magnification in 24-bit color. Computational semiautomated morphometric analysis was performed on 5× magnification images of transmural tissue sections on the basis of green color depth thresholds (ImageJ, National Institutes of Health, Bethesda, Maryland), with blinding to the diagnosis and cardiac wall. Epicardial, mid-myocardial, and endocardial zones and fat cells were defined by consensus (Figure 1A). Regions of collagen and fat were defined by color threshold, with proportions calculated by cardiac wall and tissue zone relative to tissue area.

#### Confocal microscopy analysis of post-mortem Cx43 distribution

An RVOT section from each post-mortem case underwent Cx43 immunofluorescent staining (Online Methods) to evaluate gap junction remodeling. Three transmural tissue strips of 450 μm width with intact myocardium per case were identified using 4′,6-diamidino-2-phenylindole immunofluorescence, blinded to the Cx43 signal. A Zeiss LSM-780 (Carl Zeiss Ltd., Cambridge, United Kingdom) inverted confocal microscope (20×, 0.8 numerical aperture objective lens) with sequential channel scanning (Alexa Fluor 488, 4′,6-diamidino-2-phenylindole, and cyanine Cy3 fluorescence) in a single optical plane was used. Cx43 was defined by color threshold (ImageJ). Perinuclear lipofuscin was excluded.

Morphometric analysis of Cx43 was performed as for collagen. Serial sections immediately adjacent to the Cx43-stained strip were imaged with PSR to permit correction for collagen content (Figure 1B) by dividing by the proportion representing the noncollagenous component. Adjusted and unadjusted Cx43 proportions were aggregated per subject.

### In vivo open thoracotomy mapping and ablation of RVOT

**Figure 2:**
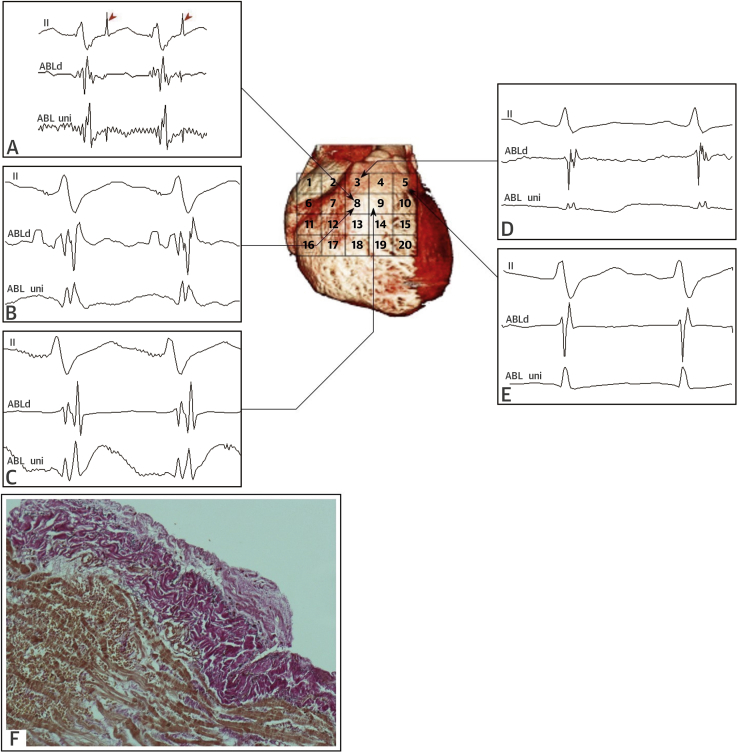
Computed Tomography Scan, Epicardial Electrograms, and Histology of RVOT of In Vivo BrS Patient Computed tomography scan of the heart **(center)** of in vivo BrS patient V2 showing an anatomical grid over the anterior RVOT. ECG lead II and a distal bipolar (0.4 mV/cm voltage scale at 30- to 300-Hz filter settings) and unipolar (5 mV/cm voltage scale at 0.05- to 300-Hz filter settings) electrogram at labeled sites are given in surrounding panels, with pacing stimuli indicated by **red arrowheads**. Abnormal fractionated electrograms are on the **(A to C)** left and normal electrograms on the **(D to E)** right. **(F)** Epicardial biopsy and histology (PSR) at the site of the abnormal electrogram shows epicardial fibrosis with focal finger-like projections of collagen into myocardium. ABL d = distal bipolar ablation catheter electrogram; ABL uni = unipolar ablation catheter electrogram; BrS = Brugada syndrome; RVOT = right ventricular outflow tract; other abbreviations as in Figure 1.

Cases V1 to V4 underwent mini-lateral thoracotomy to expose the anterior RVOT, whereas cases V5 and V6 had a midline thoracotomy. For cases V1 to V5, epicardial mapping was performed with a 3.5-mm-tip ThermoCool catheter (Biosense Webster, Diamond Bar, California) limited to the anterior RVOT (Figure 2). Radiofrequency ablations with 20- to 45-W energy were performed off pump at substrate sites identified by abnormal late and fractionated electrograms. For case V6, electroanatomical mapping was performed with the CARTO 3 System (Biosense Webster) intraoperatively, with manual confirmation of abnormal electrogram amplitudes. Cryoablation was then performed at sites of abnormal late potentials following total cardiopulmonary bypass with aorta-bicaval cannulation. The ablation endpoint for all cases was elimination of abnormal late and fractionated electrograms in the RVOT epicardium.

### Biopsy of in vivo substrate sites in the RVOT

All sites identified with abnormal electrograms were biopsied under direct vision: off-pump sampling (cases V1 to V5) was limited to small samples of epicardial surface and myocardial tissue to minimize complications; transmural biopsies were taken during heart–lung bypass in case V6. Biopsy tissue was stained with PSR.

### Clinical endpoints

In vivo BrS subjects were reviewed 1 month post-ablation and every 3 months thereafter with ICD interrogation and ECG. Ajmaline provocation was performed at 6 months for patients recruited from Bangkok.

### Research governance

The following institutional review boards approved the study: London Stanmore Research Ethics Committee; Bhumibol Adulyadej Air Force Hospital; and Yokohama Rosai Hospital. Informed consent was obtained from subjects and/or next of kin.

### Statistical analysis

Analysis was undertaken using Stata v12.1 (StataCorp LP, College Station, Texas). Natural log transformation corrected skew in measured tissue proportions of fibrosis and fat before analysis by simple and multiple regression (using independent factors for disease status, myocardial wall, and myocardial region) with robust variances; analyses are reported as odds ratios (OR). A p value ≤0.05 was considered significant.

## Results

### Post-mortem diagnosis of Brugada syndrome on familial cardiac evaluation

**Figure 3:**
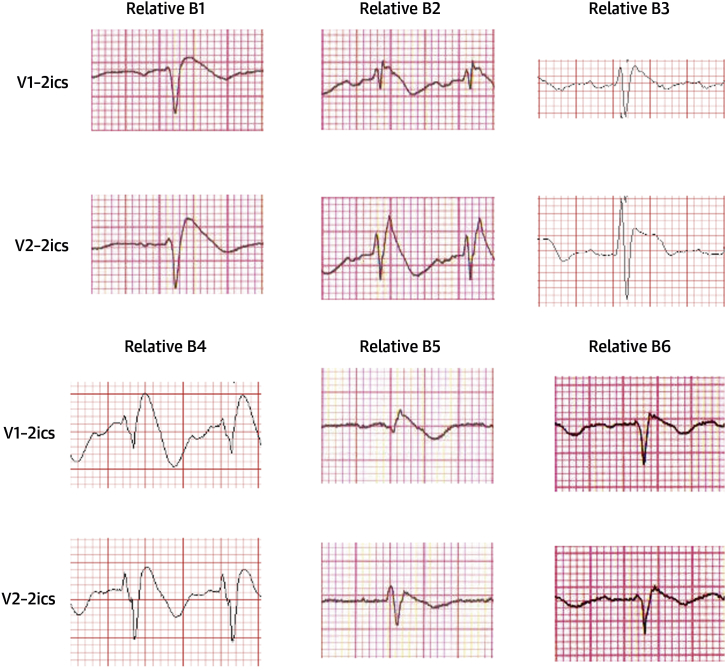
Right Precordial ECG Traces From Blood Relatives of Post-Mortem BrS Cases During Ajmaline Provocation ECG traces acquired following cranial displacement of electrode positions V1 and V2 into 2ics. 2ics = second intercostal space; other abbreviations as in Figures 1 and 2.

A mean of 3.7 first-degree blood relatives per post-mortem BrS case underwent familial evaluation, with 1.7 diagnosed with BrS on average. One relative of B4 was diagnosed with BrS on the basis of a spontaneous type 1 Brugada ECG pattern, with other relatives identified following ajmaline provocation (Figure 3). No relatives had evidence of structural or functional myocardial disease on cardiac imaging.

### Genetic mutation analysis

Five of the 6 families of post-mortem BrS cases consented to genetic analysis; 2 affected relatives of B4 were found to carry the p.Leu1462Gln mutation in *SCN5A*. Poor quality of extracted DNA prevented confirmation in B4. All in vivo cases underwent genetic testing and 2 *SCN5A* mutation carriers were identified (case V4 p.Ser528Cys and case V6 p.Leu846Arg).

### Collagen staining and myocardial architecture of the RVOT

**Figure 4:**
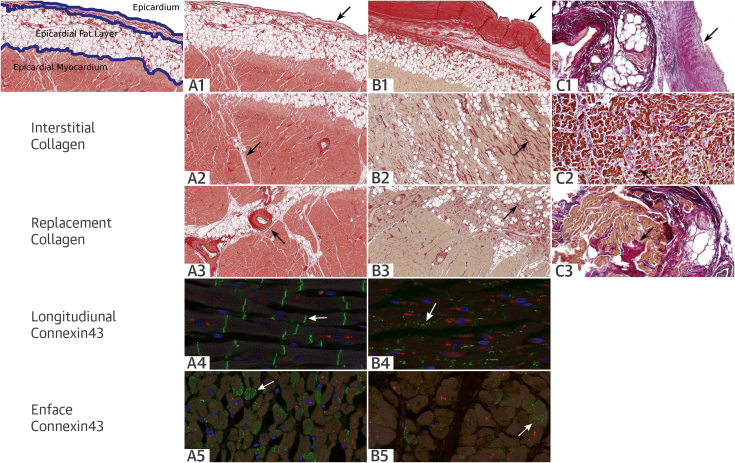
RVOT Histological Sections Stained for Collagen and Immunoconfocal Images of Cx43 Expression RVOT histological sections stained for collagen **(purple-red)** with PSR and immunoconfocal images of gap junction protein Cx43 expression (green fluorescence). Sections from **(A)** post-mortem control, **(B)** post-mortem BrS cases, and **(C)** in vivo BrS patients. **(A)** Post-mortem control. PSR: **(A1)** normal epicardial collagen thickness, with **(A2)** linear collagen between myocytes and **(A3)** around blood vessels, but no evidence of complete circumscription of myocytes by collagen. Cx43: **(A4)** normal appearance of gap junction signal concentrated to form transverse stripes, with an organized parallel orientation. **(A5)** Clusters of gap junctions in a typical ring-like formation at the intercalated disc, with large gap junctions circumscribing the periphery of the disc and smaller junctions in the inner region. **(B)** Post-mortem BrS. PSR: **(B1)** thickened epicardial collagen layer, with **(B2)** evidence of interstitial fibrosis, identified by collagen circumscribing myocytes, and **(B3)** replacement fibrosis, identified by replacement of myocytes by collagen in a region of infiltration by fat. Cx43: **(B4)** notable dispersion of the signal along the axis of the cell and **(B5)** sparse junctional plaque with an ill-defined border. **(C)** In vivo BrS. PSR: **(C1)** thickened epicardial collagen layer with **(C2)** evidence of interstitial fibrosis, identified by collagen circumscribing myocytes, and **(C3)** replacement fibrosis, identified by replacement of myocytes by collagen. Abbreviations as in Figures 1 and 2.

Myocardial collagen in the control group was seen in the epicardial surface and around blood vessels. Linear collagen was distributed parallel to myocytes, but did not surround the individual myocytes (Figure 4A2 and 4A3). This collagen distribution pattern is normal in the RV.

In the post-mortem BrS group, there was an appearance of increased epicardial surface collagen that was thicker than that in control hearts, indicating epicardial fibrosis (Figure 4B1). There was infiltration of the epicardial surface fibrosis into the underlying epicardial myocardium, with individual myocytes surrounded by collagen, which was considered interstitial myocardial fibrosis (Figure 4B2). There was also evidence of replacement of myocytes by collagen, focal replacement fibrosis, admixed with fat in the epicardial myocardium (Figure 4B3). The in vivo tissue samples taken in the regions of late potentials showed similar epicardial and myocardial fibrosis patterns (Figure 4C1 to 4C3). The epicardial fibrosis appeared to be separated from the underlying myocardium by fat in some sections, whereas in others, it infiltrated directly into the underlying myocardium.

### Morphometric analysis of post-mortem collagen by PSR

**Figure 5:**
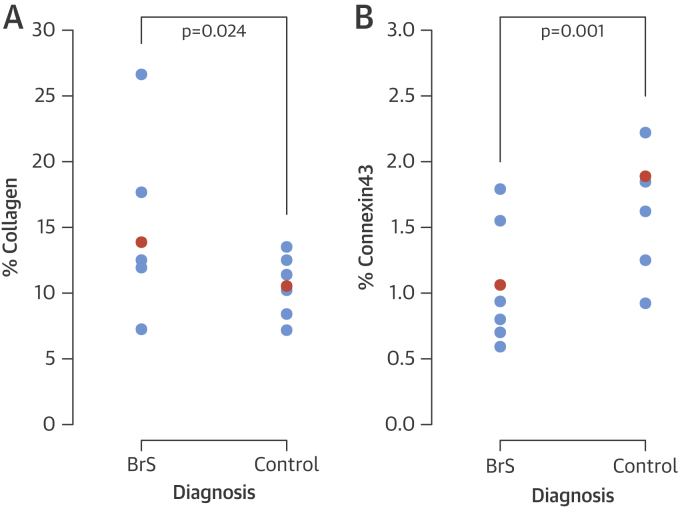
Scatterplot of Collagen and Cx43 Quantification in the Epicardial Myocardium of the Right Ventricular Outflow Tract of BrS and Control Post-Mortem Cases **(A)** PSR quantification of collagen content and **(B)** immunofluorescence quantification of Cx43. **Orange data points** represent distribution means. **Blue data points** represent individual cases and controls. Abbreviations as in Figures 2 and 3.

The BrS cohort had greater collagen content than control hearts, with maximal differences seen in the RVOT epicardium (13.9% vs. 10.5%; p = 0.024) (Figure 5A). Multivariable analysis (Table 2) identified that the diagnosis of BrS was associated with an OR of 1.42 (p = 0.026) for collagen proportion, regardless of the cardiac chamber.

Control hearts and cases also showed similar patterns of collagen distribution, but this was greater in cases. The RVOT (OR: 1.98; p = 0.003) and RV (OR: 1.66; p = 0.020) walls had higher collagen content in comparison with the LV, irrespective of diagnosis. Similarly, a gradient of decreasing collagen content was seen from the epicardial to endocardial zones (OR: 2.00; p = 0.001) in all chambers.

### Morphometric analysis of post-mortem fat cells

Regression analysis for the proportion of fat content in the myocardium showed no significant difference between BrS and control hearts (p = 0.133).

### Post-mortem Cx43 signal distribution and quantification

In control myocardial tissue, Cx43 localized to the intercalated disc (Figure 4A4 and 4A5). BrS cases showed a reduced Cx43 signal and a decreased punctate pattern in the intercalated disc (Figure 4B4 and 4B5).

BrS cases had reduced Cx43 signal in the RVOT compared with control hearts (OR: 0.59; p = 0.001) (Figure 5B, Table 3), even following correction for collagen content (OR: 0.58; p = 0.036). No significant difference was observed between myocardial zones of the RVOT (p = 0.476).

### Clinical outcomes

The mean radiofrequency ablation time was 14 ± 6 min per in vivo BrS case; no surgical complications occurred. In the 5 patients who underwent radiofrequency ablation, fractionated electrograms disappeared immediately, with a drastic reduction of ventricular electrograms after radiofrequency was turned off. The ECG pattern normalized (i.e., reversion from type 1 Brugada ECG pattern) within a week in all cases, and a negative ajmaline test was seen in those who underwent subsequent provocation 3 months later (n = 5 of 6). No further ventricular tachycardia (VT) or ventricular fibrillation (VF) episodes were seen during the follow-up period (mean 24.6 ± 9.7 months, median 25 months), and quinidine therapy was not required.

## Discussion

This study systematically describes increased collagen content in the RVOT that shows epicardial surface and intramyocardial fibrosis, as well as diminished gap junction protein expression. In vivo human evidence of conduction delay in the RVOT was associated with similar patterns of fibrosis, corroborating the post-mortem findings (Central Illustration). Ablation at these sites eliminated the type 1 ECG pattern with successful suppression of VT/VF recurrence, giving support to the hypothesis that conduction delay is responsible for the BrS phenotype.

### Myocardial fibrosis

Despite the a priori exclusion at expert autopsy of overt structural abnormalities in SADS cases, the diagnosis of BrS was associated with increased collagen content in all ventricular walls. This was over and above the normal collagen seen in age- and sex-matched control hearts. In addition, the in vivo cases all had normal cardiac imaging, including computed tomography/magnetic resonance imaging, as well as macroscopically normal hearts on direct visualization during thoracotomy. These cases, therefore, represent minimally structurally perturbed candidates for the diagnosis of BrS, yet they showed distinctive patterns of fibrosis. This reveals the limitations of current imaging technology for detecting subtle changes in the myocardium that can still give rise to physiologically detectable changes.

We have identified previously that one-third of unexplained SCDs with idiopathic fibrosis and/or hypertrophy had familial diagnoses of BrS (12). LV and RV free-wall, age-related fibrosis has also been seen in mouse models of BrS 21, 22. In addition, we identified epicardial and intramyocardial fibrosis at the site of epicardial late potentials in the RVOT of BrS patients. A detailed study of a single patient with BrS who underwent transplantation has previously colocalized interstitial fibrosis with conduction delay (14). Moreover, murine models of BrS, including epicardial electrophysiological study of Langendorff perfused hearts, have shown RVOT pathology: increased collagen; delayed conduction; and a propensity for ventricular arrhythmia with programmed stimulation in the RVOT (23). It is therefore plausible that BrS may reflect a generalized disease of myocardial architecture, with baseline properties of the RVOT predisposing it to fibrosis, which is likely to underlie the condition and arrhythmic risk (24). Interestingly, although fibrosis and conduction delay have been identified in carriers of *SCN5A* mutations (25), all cases demonstrated some evidence of fibrosis, whether they harbored an *SCN5A* mutation or not. The reported increase in profibrotic markers secondary to sodium channel inactivation, independent of messenger ribonucleic acid expression, suggests that fibrosis may be a feature irrespective of mutation status (26).

### Fat infiltration of myocardium

No significant difference in fat content was observed between BrS cases and control hearts. In contrast, transmural fat infiltration in the absence of fibrosis predominated in Italian post-mortem cases with the Brugada ECG pattern (8). This difference may reflect the inclusion of patients with overt antemortem and post-mortem features of arrhythmogenic right ventricular cardiomyopathy in the Italian study without suitable age- and sex-matched controls.

### Significance of Cx43

The Cx43 signal was diminished in BrS compared with the control myocardium. This raises the possibility that changes at the intercalated disc that affect Cx43 expression may cause cardiomyocyte electrical uncoupling, and therefore, may be important in the pathogenesis of BrS. Royer et al. (21) describe diminished Cx43 expression in the *scn5a*-knockout mouse model’s myocardium, which is a clear correlation with the human phenotype.

### Open thoracotomy catheter ablation

As previously reported (10), abolition of the type 1 ECG and suppression of VT/VF episodes in a high-risk BrS patient cohort were seen following epicardial ablation at sites of late potentials in the RVOT. To our knowledge, this study reports, for the first time, a surgical approach with either midline or mini-lateral thoracotomy to access the epicardial surface of the RVOT for ablation.

### Depolarization versus repolarization

Our findings reinforce other human studies that have identified conduction delay in the RVOT in BrS in vivo 10, 27, 28, 29, 30. Two of these studies used noncontact intracardiac mapping or noninvasive ECG imaging and proposed additional repolarization abnormalities 27, 30. We have correlated directly acquired delayed, prolonged, and fragmented epicardial electrograms and histopathological evidence for fibrosis that support depolarization delay as the primary substrate.

### Study limitations

Subject recruitment was limited by the rarity of thoracotomy in BrS patients and the availability of whole hearts post-mortem in which families were diagnosed with BrS. Thus, our cohorts represent a unique collection. Both control and case hearts went through similar processing after death, with an approximate 24- to 48-h delay before fixation and an intervening period of refrigeration. We were unable to establish more accurate timing.

The etiology of death in the 6 BrS post-mortem cases was established by identifying BrS in blood relatives in the absence of alternative explanations. This methodology forms the basis of internationally accepted guidelines for the diagnosis of genetic disorders in unexplained SCD and BrS 1, 3. However, we recognize that without previous ECG evidence, we cannot be absolutely certain of the diagnosis. Nonetheless, it is a reasonable assumption, as the deceased young person does, at a minimum, have a 50% chance of having the same diagnosis. The chance of any other diagnosis is much smaller. In addition, the finding of 1 *SCN5A* mutation in the 5 families tested is consistent with the established prevalence of 20% in BrS (5). Retrospective investigation by molecular autopsy was not possible in our cases, although the absence of a mutation would not exclude BrS due to the low molecular genetic yield (5).

Our study only included symptomatic BrS cases. Thus, our observations may reflect a biased population of high-risk subjects. However, myocardial fibrosis has also been identified in low-risk living patients on magnetic resonance imaging 31, 32 and histopathology (33).

## Conclusions

BrS, in the absence of overt structural or functional abnormalities, is unequivocally associated with increased collagen, fibrosis, and reduced gap junction expression in the RVOT. Myocardial late potentials indicative of the arrhythmic substrate anatomically collocate with fibrosis in the RVOT of BrS subjects. Therefore, it is plausible that BrS represents a disease of myocardial architecture and cardiomyocyte electrical coupling in the RVOT. The reduction in arrhythmic burden and reversal of electrocardiographic signature of BrS following ablation at these sites supports our hypothesis that these myocardial changes result in discontinuity of cardiac conduction responsible for arrhythmogenesis. These data are the strongest yet to support the depolarization theory of the pathogenesis of BrS 29, 34.

Perspectives **COMPETENCY IN MEDICAL KNOWLEDGE:** Two main theories have been proposed to explain the pathophysiology of BrS: 1) that it is due to either dispersed repolarization; or 2) to abnormal depolarization due to conduction delay. Tissue from cases of SCD due to BrS without evident structural disease exhibits increased collagen throughout the heart and fibrosis, as well as reduced gap junction signaling protein Cx43 in the RVOTs of those with BrS compared with tissue from victims of noncardiac death. Myocardial biopsies before epicardial ablation also display fibrosis at sites of delayed activation in patients with BrS. These data support the depolarization hypothesis.

**TRANSLATIONAL OUTLOOK:** Future studies should address the roles of quantification of fibrosis and gap junction proteins in the diagnosis of and risk stratification for SCD among patients with known or suspected BrS and identify the predictors and determinants of these structural abnormalities.

## Figures and Tables

**Central Illustration:**
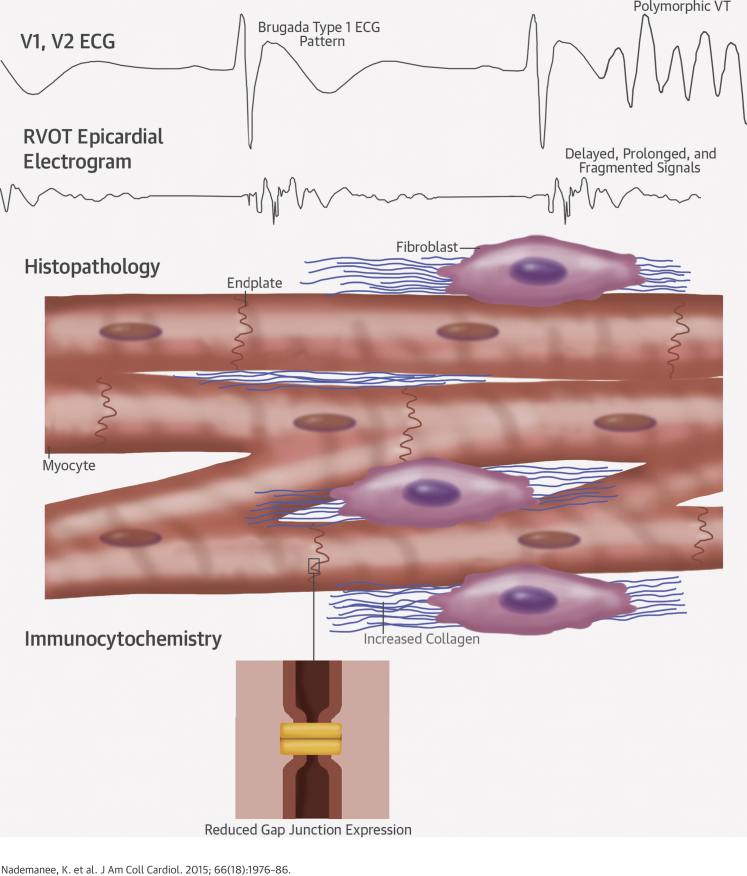
Pathophysiology of Brugada Syndrome: Conduction Delay Due to Fibrosis and Connexin-43 Abnormalities Conduction delay in the right ventricular outflow tract (RVOT) is caused by myocyte electrical uncoupling due to a reduction in connexin-43 at endplates and subtle interstitial and replacement fibrosis. As a result, epicardial electrograms are abnormal, slowed, and fragmented. This provides the substrate for the Brugada type 1 electrocardiographic (ECG) pattern, re-entry, and the generation of polymorphic ventricular tachycardia (VT) and ventricular fibrillation.

**Table 1:** Demographic Data, Familial Evaluation Results, and Index Presentation for the Included Post-Mortem BrS, Post-Mortem Control, and In Vivo BrS Cases

Case	Sex	Age (yrs)	Index Presentation	Clinical Abnormality	Cardiac Morphology	Relatives Evaluated	Relatives Affected
Post-mortem BrS cohort							
B1	M	15	SCD in sleep	Diagnosis in relative	Normal	2	2
B2	M	18	SCD in sleep	Diagnosis in relative	Normal	4	1
B3	M	19	SCD in sleep	Diagnosis in relative	Normal	5	1
B4	M	23	SCD with exercise	Diagnosis in relative	Tunneled RCA	3	2
B5	M	24	SCD in sleep	Diagnosis in relative	Atrial septal defect	3	1
B6	M	40	SCD with minimal activity	Diagnosis in relative	Normal	5	3
Post-mortem control cohort							
C1	M	17	RTA	None	Normal	—	—
C2	M	18	RTA	None	Normal	—	—
C3	M	22	Suicide	None	Normal	—	—
C4	M	22	RTA	None	Normal	—	—
C5	M	22	RTA	None	Normal	—	—
C6	M	37	Homicide	None	Normal	—	—
In vivo BrS cohort							
V1	M	48	Multiple syncope	Spontaneous type 1 ECG	Normal	—	—
V2	M	28	Multiple syncope	Ajmaline-provoked type 1 ECG	Normal	—	—
V3	M	59	VF arrest	Spontaneous type 1 ECG	Normal	—	—
V4	M	29	VF arrest with fever	Spontaneous type 1 ECG	Normal	—	—
V5	M	47	Syncope	Spontaneous Type 1 ECG	Normal	—	—
V6	M	27	Multiple syncope	Spontaneous type 1 ECG	Normal	—	—

BrS = Brugada syndrome; ECG = electrocardiogram; M = male; RCA = right coronary artery; RTA = road traffic accident; SCD = sudden cardiac death; VF = ventricular fibrillation.

**Table 2:** Univariable and Multivariate Regression Analysis of Proportional Collagen Content, as Evaluated by Morphometric Analysis of PSR Staining in BrS Cases Versus Control Hearts

Variable	BrS vs. Control Hearts			
Univariable Analysis		Multivariate Analysis	
OR (95% CI)	p Value	OR (95% CI)	p Value
Disease	1.42 (1.06–1.90)	0.024	1.42 (1.05–191)	0.026
LV	1.00	N/A	1.00	N/A
RV	1.66 (1.11–2.50)	0.019	1.66 (1.10–2.51)	0.020
RVOT	1.98 (1.34–2.91)	0.003	1.98 (1.33–2.93)	0.003
Endo	1.00	N/A	1.00	N/A
Mid	1.27 (1.02–1.58)	0.033	1.27 (1.02–1.58)	0.035
Epi	2.00 (1.46–2.73)	<0.001	2.00 (1.45–2.74)	0.001

BrS = Brugada syndrome; CI = confidence interval; Endo = endocardium; Epi = epicardium; LV = left ventricle; Mid = mid-myocardium; OR = odds ratio; PSR = picrosirius red; RV = right ventricle; RVOT = right ventricular outflow tract.

**Table 3:** Multivariable Regression Analysis of Proportional Connexin43 Content in BrS Post-Mortem Cases Versus Control Hearts

Variable	BrS vs. Control Hearts	
OR (95% CI)	p Value
Disease	0.59 (0.44–0.79)	0.001
Endocardium	1.00	N/A
Mid-myocardium	0.97 (0.64–1.49)	0.897
Epicardium	1.16 (0.76–1.78)	0.476
Disease (corrected for collagen)	0.58 (0.36–0.96)	0.036

Expression according to zone and after correction for collagen content is also shown.

Abbreviations as in Table 2.

## References

[1] Priori S.G., Wilde A.A., Horie M. (2013). Executive summary: HRS/EHRA/APHRS expert consensus statement on the diagnosis and management of patients with inherited primary arrhythmia syndromes. Europace.

[2] Brugada P., Brugada J. (1992). Right bundle branch block, persistent ST segment elevation and sudden cardiac death: a distinct clinical and electrocardiographic syndrome. A multicenter report. J Am Coll Cardiol.

[3] Behr E., Wood D.A., Wright M., for the Sudden Arrhythmic Death Syndrome (SADS) Steering Group (2003). Cardiological assessment of first-degree relatives in sudden arrhythmic death syndrome. Lancet.

[4] Raju H., Behr E.R. (2013). Unexplained sudden death, focussing on genetics and family phenotyping. Curr Opin Cardiol.

[5] Hedley P.L., Jørgensen P., Schlamowitz S. (2009). The genetic basis of Brugada syndrome: a mutation update. Hum Mutat.

[6] Yan G.X., Antzelevitch C. (1999). Cellular basis for the Brugada syndrome and other mechanisms of arrhythmogenesis associated with ST-segment elevation. Circulation.

[7] Corrado D., Nava A., Buja G. (1996). Familial cardiomyopathy underlies syndrome of right bundle branch block, ST segment elevation and sudden death. J Am Coll Cardiol.

[8] Corrado D., Basso C., Buja G. (2001). Right bundle branch block, right precordial ST-segment elevation, and sudden death in young people. Circulation.

[9] Ohkubo K., Watanabe I., Okumura Y. (2010). Right ventricular histological substrate and conduction delay in patients with Brugada syndrome. Int Heart J.

[10] Nademanee K., Veerakul G., Chandanamattha P. (2011). Prevention of ventricular fibrillation episodes in Brugada syndrome by catheter ablation over the anterior right ventricular outflow tract epicardium. Circulation.

[11] Morita H., Zipes D.P., Morita S.T. (2009). Epicardial ablation eliminates ventricular arrhythmias in an experimental model of Brugada syndrome. Heart Rhythm.

[12] Papadakis M., Raju H., Behr E.R. (2013). Sudden cardiac death with autopsy findings of uncertain significance: potential for erroneous interpretation. Circ Arrhythm Electrophysiol.

[13] Frustaci A., Priori S.G., Pieroni M. (2005). Cardiac histological substrate in patients with clinical phenotype of Brugada syndrome. Circulation.

[14] Coronel R., Casini S., Koopmann T.T. (2005). Right ventricular fibrosis and conduction delay in a patient with clinical signs of Brugada syndrome: a combined electrophysiological, genetic, histopathologic, and computational study. Circulation.

[15] Wilde A.A.M., Postema P.G., Di Diego J.M. (2010). The pathophysiological mechanism underlying Brugada syndrome: depolarization versus repolarization. J Mol Cell Cardiol.

[16] Waldo K.L., Lo C.W., Kirby M.L. (1999). Connexin 43 expression reflects neural crest patterns during cardiovascular development. Dev Biol.

[17] Elizari M.V., Levi R., Acunzo R.S. (2007). Abnormal expression of cardiac neural crest cells in heart development: a different hypothesis for the etiopathogenesis of Brugada syndrome. Heart Rhythm.

[18] Raju H., Papadakis M., Govindan M. (2011). Low prevalence of risk markers in cases of sudden death due to Brugada syndrome: relevance to risk stratification in Brugada syndrome. J Am Coll Cardiol.

[19] Govindan M., Batchvarov V.N., Raju H. (2010). Utility of high and standard right precordial leads during ajmaline testing for the diagnosis of Brugada syndrome. Heart.

[20] Basso C., Burke M., Fornes P., for the Association for European Cardiovascular Pathology (2010). Guidelines for autopsy investigation of sudden cardiac death. Pathologica.

[21] Royer A., van Veen T.A.B., Le Bouter S. (2005). Mouse model of *SCN5A*-linked hereditary Lenègre’s disease: age-related conduction slowing and myocardial fibrosis. Circulation.

[22] Jeevaratnam K., Rewbury R., Zhang Y. (2012). Frequency distribution analysis of activation times and regional fibrosis in murine *Scn5a*^*+/-*^ hearts: the effects of ageing and sex. Mech Ageing Dev.

[23] Zhang Y., Guzadhur L., Jeevaratnam K. (2014). Arrhythmic substrate, slowed propagation and increased dispersion in conduction direction in the right ventricular outflow tract of murine *Scn5a*+/- hearts. Acta Physiol (Oxf).

[24] Hoogendijk M.G., Potse M., Linnenbank A.C. (2010). Mechanism of right precordial ST-segment elevation in structural heart disease: excitation failure by current-to-load mismatch. Heart Rhythm.

[25] Meregalli P.G., Tan H.L., Probst V. (2009). Type of *SCN5A* mutation determines clinical severity and degree of conduction slowing in loss-of-function sodium channelopathies. Heart Rhythm.

[26] Hao X., Zhang Y., Zhang X. (2011). TGF-β1-mediated fibrosis and ion channel remodeling are key mechanisms in producing the sinus node dysfunction associated with *SCN5A* deficiency and aging. Circ Arrhythm Electrophysiol.

[27] Lambiase P.D., Ahmed A.K., Ciaccio E.J. (2009). High-density substrate mapping in Brugada syndrome: combined role of conduction and repolarization heterogeneities in arrhythmogenesis. Circulation.

[28] Sacher F., Jesel L., Jais P. (2014). Insight into the mechanism of Brugada syndrome: epicardial substrate and modification during ajmaline testing. Heart Rhythm.

[29] Postema P.G., van Dessel P.F.H.M., de Bakker J.M.T. (2008). Slow and discontinuous conduction conspire in Brugada syndrome: a right ventricular mapping and stimulation study. Circ Arrhythm Electrophysiol.

[30] Zhang J., Sacher F., Hoffmayer K. (2015). Cardiac electrophysiological substrate underlying the ECG phenotype and electrogram abnormalities in Brugada syndrome patients. Circulation.

[31] Papavassiliu T., Wolpert C., Flüchter S. (2004). Magnetic resonance imaging findings in patients with Brugada syndrome. J Cardiovasc Electrophysiol.

[32] Van Hoorn F., Campian M.E., Spijkerboer A. (2012). *SCN5A* mutations in Brugada syndrome are associated with increased cardiac dimensions and reduced contractility. PloS One.

[33] Zumhagen S., Spieker T., Rolinck J. (2009). Absence of pathognomonic or inflammatory patterns in cardiac biopsies from patients with Brugada syndrome. Circ Arrhythm Electrophysiol.

[34] Hoogendijk M.G., Opthof T., Postema P.G. (2010). The Brugada ECG pattern: a marker of channelopathy, structural heart disease, or neither? Toward a unifying mechanism of the Brugada syndrome. Circ Arrhythm Electrophysiol.

