# Supplementary material for: Fibrosis, Connexin-43, and Conduction Abnormalities in the Brugada Syndrome
Source: J Am Coll Cardiol. 2015 Nov 3;66(18):1976–86. doi: 10.1016/j.jacc.2015.08.862 (PMC4631798; doi:10.1016/j.jacc.2015.08.862)
Supplement: Online Methods [file mmc1.docx]

# SUPPLEMENTARY APPENDIX

Table of Contents

SUPPLEMENTARY APPENDIX 1

eMETHODS 2

Familial Cardiological Evaluation 2

Diagnosis of Brugada Syndrome 2

Preparation of Histologic Slides 3

Cx43 Quantification 3

REFERENCES 5

## Online METHODS

### Familial Cardiological Evaluation

Blood relatives of post-mortem Brugada syndrome (BrS) cases underwent comprehensive non-invasive cardiological evaluation to confirm the underlying diagnosis.^1^ This included investigation with resting, 24-hour ambulatory and exercise ECGs, echocardiography and cardiac magnetic resonance imaging (MRI), in addition to ajmaline provocation-testing. A diagnosis of BrS in a first-degree relative following sudden cardiac death and negative autopsy (SADS) established BrS as the cause of death in the post-mortem cohort.^1^

### Diagnosis of Brugada Syndrome

Resting ECG and ajmaline provocation-testing were performed using standard 12-lead in addition to V1 and V2 cranially displaced into the 3rd and/or 2nd intercostal space. Ajmaline was administered intravenously over 5 minutes to a target dose of 1mg/kg or until appearance of the type 1 Brugada ECG pattern.^2^

A diagnosis of BrS was based on a spontaneous or ajmaline-provoked type 1 Brugada ECG pattern in 2 or more right ventricular leads (V1, V2 and V3 located in 2nd, 3rd or 4th intercostal spaces) in the absence of structural or functional cardiac disease on other cardiac investigations.^3,4^

### Preparation of Histologic Slides

Tissues were fixed in a solution of 10% neutral buffered formalin, embedded in paraffin and sectioned at a thickness of 5μm.

Immunofluorescence

Myocardial tissue was previously fixed in a solution of 10% neutral buffered formalin, embedded in paraffin and sectioned at a thickness of 5μm. The tissue was hydrated following washes in xylene through a series of alcohols followed by water. After blocking with 1% BSA in PBS (1 hour), sections were immediately incubated with anti-connexin43 antibody (0.2 μg/ml, Sigma-Aldrich, UK) and left overnight at 4C. Following washes with PBS, sections were incubated with Alexa 488-conjugated anti-rabbit whole IgG for 1 hour at room temperature and briefly (2 minutes) with nuclear dye DAPI (0.2 μg/ml) (Vectorlabs, California, US) before washing in PBS and mounting in Citiflour AF (Citifluour Ltd., London, UK).

### Cx43 Quantification

Images were recorded using a Zeiss LSM-780 inverted confocal microscope. A low power overview image in the Dapi channel was used to select 3 representative strips spanning the myocardium to avoid any bias with the acquisition of the Cx43 signal channel. Analysis was performed using a 20x 0.8 na objective lens using sequential channel scanning (Alexa 488, DAPI and CY3 fluorescence) in a single optical plane. Each strip consisted of sequential images taken from the epicardium to the endocardium with a width of 450µm and length up to 1cm. A single montage was created from overlapping images using ImageJ. True Cx43 pixels were defined in the 256-leveled green channel. Isolated pixels were excluded from the analysis. Perinuclear lipofuschin was excluded by distinguishing an orange-red appearance. Cx43 signal was expressed as a proportion of the signal density to tissue area measured for epicardial, mid-myocardial and endocardial zones as per morphometric analysis. In order to more accurately compare proportions of myocyte Cx43 signal, serial sections immediately adjacent to the Cx43 stained section from each patient/group were stained with picrosirius red and imaged using a 10x objective lens on an Axio Observer Inverted Widefield Microscope (Carl Zeiss Microscopy). The total tissue area was then adjusted for the proportion of collagen in order to obtain a signal to myocyte ratio (Figure 3). Cx43 proportions were then aggregated per individual and compared between cases of BrS and controls.

## REFERENCES

1. Raju H, Papadakis M, Govindan M, et al. Low prevalence of risk markers in cases of sudden death due to Brugada syndrome relevance to risk stratification in Brugada syndrome. *J Am Coll Cardiol* 2011; **57**: 2340–5.

2. Govindan M, Batchvarov VN, Raju H, et al. Utility of high and standard right precordial leads during ajmaline testing for the diagnosis of Brugada syndrome. *Heart* 2010; **96**: 1904–8.

3. Priori SG, Wilde AA, Horie M, et al. Executive summary: HRS/EHRA/APHRS expert consensus statement on the diagnosis and management of patients with inherited primary arrhythmia syndromes. *Europace*. 2013; **15**: 1389–406.

4. Antzelevitch C, Brugada P, Borggrefe M, et al. Brugada syndrome: report of the second consensus conference: endorsed by the Heart Rhythm Society and the European Heart Rhythm Association. *Circulation* 2005; **111**: 659–70.
